# Ascochyta blight in North Dakota field pea: the pathogen complex and its fungicide sensitivity

**DOI:** 10.3389/fpls.2023.1165269

**Published:** 2023-08-02

**Authors:** Dimitri L. Fonseka, Samuel G. Markell, Marcio L. Zaccaron, Malaika K. Ebert, Julie S. Pasche

**Affiliations:** Department of Plant Pathology, North Dakota State University, Fargo, ND, United States

**Keywords:** Ascochyta blight, *Didymella pinodes*, *Ascochyta pisi*, pyraclostrobin, prothioconazole, fungicide resistance, *Pisum sativum* L.

## Abstract

Worldwide, Ascochyta blight is caused by a complex of host-specific fungal pathogens, including *Ascochyta pisi*, *Didymella pinodes*, and *Didymella pinodella*. The application of foliar fungicides is often necessary for disease management, but a better understanding of pathogen prevalence, aggressiveness, and fungicide sensitivity is needed to optimize control. Leaf and stem samples were obtained from 56 field pea production fields in 14 counties in North Dakota from 2017 to 2020 and isolates were collected from lesions characteristic of Ascochyta blight. Based on fungal characteristics and sequencing the ITS1-5.8S-ITS2 region, 73% of isolates were confirmed to be *D. pinodes* (n = 177) and 27% were *A. pisi* (n = 65). Across pathogens, aggressiveness was similar among some isolates in greenhouse assays. The *in vitro* pyraclostrobin sensitivity of all *D. pinodes* isolates collected from 2017 to 2020 was lower than that of the three baseline isolates. Sensitivity of 91% of *A. pisi* isolates collected in 2019 and 2020 was lower than the sensitivity of two known sensitive isolates. Resistance factors (Rf) from mean EC_50_ values of pyraclostrobin baseline/known sensitive isolates to isolates collected from 2017 to 2020 ranged from 2 to 1,429 for *D. pinodes* and 1 to 209 for *A. pisi*. *In vitro* prothioconazole sensitivity of 91% of *D. pinodes* isolates collected from 2017 to 2020 was lower than the sensitivity of the baseline isolates and 98% of *A. pisi* isolates collected from 2019 to 2020 was lower than the sensitivity of the known sensitive isolates. Prothioconazole Rf ranged from 1 to 338 for *D. pinodes* and 1 to 127 for *A. pisi*. Based on *in vitro* results, 92% of *D. pinodes* and 98% of *A. pisi* isolates collected displayed reduced-sensitivity/resistance to both fungicides when compared to baseline/known sensitive isolates. Disease control under greenhouse conditions of both pathogens provided by both fungicides was significantly lower in isolates determined to be reduced-sensitive or resistant in *in vitro* assays when compared to sensitive. Results reported here reinforce growers desperate need of alternative fungicides and/or management tools to fight Ascochyta blight in North Dakota and neighboring regions.

## 1 Introduction

Field pea (*Pisum sativum* L.) production has increased substantially in North America over the last two decades. North Dakota ranks second in field pea production in the United States behind Montana, with 98,000 ha harvested in 2021 (United States Department of Agriculture National Agriculture Statistics Service USDA-NASS, 2022). Ascochyta blight is among the most important diseases of field pea in North America (Tivoli and Baninza, 2007) and disease progression is favored by cool, wet weather from bloom until mid-pod development. In peas, Ascochyta blight is caused by a complex of host-specific fungal pathogens (Tivoli and Baninza, 2007; Davidson et al., 2009; Davidson et al., 2021). *Ascochyta pisi* Lib, *Didymella pinodes* (Berk. & A. Bloxam) Petr., and *Didymella pinodella* (L.K. Jones) Qian Chen & L. Cai have been documented as infecting field pea in the northern Great Plains region of North America (Owati et al., 2019). These three fungal pathogens have been associated with the disease in many field pea producing countries including the USA (Peever et al., 2007), Australia (Ali et al., 1978), Canada (Beasse et al., 1999), China (Liu et al., 2016), France (Le May et al., 2018), and Spain (Barilli et al., 2016). *Phoma koolunga* (Davidson et al., 2009), *Phoma herbarum* (Li et al., 2011), *Boerema exigua* var. *exigua* (Li et al., 2012), and *Phoma glomerata* (Tran et al., 2014) are associated with the disease complex, also known as black spot of field pea in Australia. *P. koolunga* has been widely distributed in Australia and has become an important component of the Ascochyta blight complex of field pea (Davidson et al., 2012; Tran et al., 2014; Keirnan et al., 2020).

**Figure 1:**
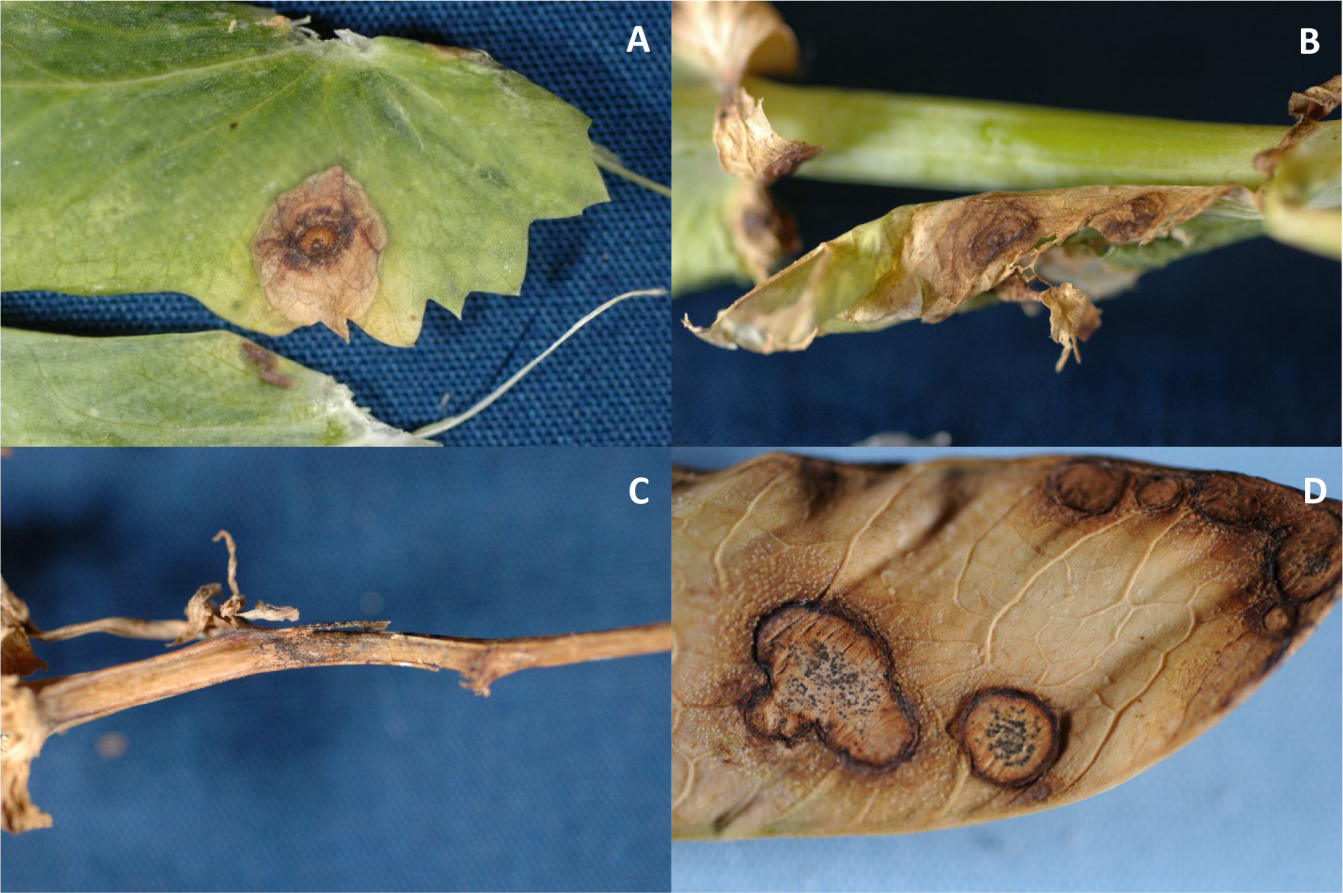
Symptoms of Ascochyta blight on leaves **(A, B)**, a stem **(C)**, and pod **(D)** (Photos: S. G. Markell).

Ascochyta blight pathogens infect all above-ground parts of the pea plant and it is difficult to distinguish among symptoms caused by these fungi (Skoglund et al., 2011; Barbetti et al., 2021). Symptoms include purplish black to brown lesion on stems, leaves, and pods (**Figure 1**). *D. pinodella* most typically causes foot rot and has been the most frequently isolated pathogen from pea roots in Sweden and Denmark (Persson et al., 1997; Tivoli and Baninza, 2007). Seed quality and quantity are reduced by Ascochyta blight through seed discoloration and deceleration of seed development, respectively. Ascochyta blight can be misdiagnosed as bacterial blight (*Pseudomonas syringae* pv. *pisi*) or Septoria blight (*Septoria pisi*), both of which can be observed with Ascochyta blight (Barbetti et al., 2021). *D. pinodes* has been reported as the predominant and most aggressive pathogen, causing up to 70% yield loss; therefore, this species has been the focus among the Ascochyta blight pathogens (Tivoli and Baninza, 2007). *A. pisi* was the predominant pathogen recovered from seeds collected in Montana and has been reported as a pathogen of field peas in South Dakota (Mathew et al., 2010; Owati et al., 2020); however, causal agents of Ascochyta blight of field peas in North Dakota have not been reported.

The fungi that cause Ascochyta blight in North America overwinter in the soil, on debris, and can be seed-transmitted (Skoglund et al., 2011; Barilli et al., 2016). Air-borne ascospores of *D. pinodes* develop from pseudothecia on infected stubble, can travel long distances via wind, and can place even new fields at risk (Tivoli and Baninza, 2007; Schmidt, 2021). Pycnidiospores (conidia from pycnidia) of *A. pisi* and *D. pinodella* are spread to other plants via rain splash (Davidson et al., 2021). *D. pinodes* and *D. pinodella* produce chlamydospores while *A. pisi* does not. In the initial description, *D. pisi* was determined to have a longer latent period and caused less severe infections when compared to *P. pinodes* and *A. pinodella* in greenhouse evaluations (Chilvers et al., 2009). Subsequent research reported that isolates of *P. pinodes* were more aggressive in the greenhouse than isolates of *D. pinodella*, *D. pisi*, and *Phoma* spp. (Owati et al., 2020).

Effective disease management requires an integrated approach including crop rotation, use of pathogen-free certified seed, and fungicide seed treatments. Application of foliar fungicides should initiate at early flowering if the canopy is dense and cool, wet weather persists. Typically, a single fungicide application can provide adequate disease reductions if disease pressure is low (Schmidt, 2021). A second fungicide application 10- to 14-days after the first is warranted if symptoms “spread upward” in the crop canopy and favorable conditions persist. The use of locally systemic and translaminar fungicides has increased recently due to the importance of the crop in the northern Great Plains region and high disease pressure present (Schmidt, 2021). Five quinone outside inhibitor (QoI; Fungicide Resistance Action Committee [FRAC] code 11), succinate dehydrogenase inhibitor (SDHI; FRAC code 7), and demethylation inhibitor (DMI; FRAC code 3) fungicides are currently labeled as solo or pre-mixed products for the management of Ascochyta blight of field pea in North Dakota (Friskop et al., 2022).

QoI fungicides play an important role in the control of many fungi and fungal-like organisms. QoI fungicides have a single-site mode of action, interfering with electron transfer at the outer quinol oxidation site (Qo) of the cytochrome bc_1_ complex, also known as the complex III of the mitochondrial respiration chain (Bartlett et al., 2002). QoI fungicides have been classified as being high-risk for the development of resistance and field reduced-sensitivity/resistance has been reported in over 50 fungal pathogens (Pasche et al., 2004), Ishii, 2008; Bolton et al., 2013 Reimann and Deising, 2005), including. *Ascochyta rabiei* in commercial chickpea production systems in the prairie provinces of Canada and the northern Great Plains region of the US (Chang et al., 2007; Wise et al., 2009).

Resistance to QoI fungicides in *D. pinodes* has been reported in Alberta and Saskatchewan, likely due to increased selection pressure caused by sequential applications of the QoI pyraclostrobin (Headline ^®^, BASF Corporation, RTP, NC, USA) in intensive field pea production systems (Bowness et al., 2016). In that study, 6% of *D. pinodes* isolates collected in 2010 and 2011 demonstrated high levels of insensitivity to the fungicide: nine from central Alberta, five from northern Alberta, and five from south central Saskatchewan (Bowness, 2013). The 19 *D. pinodes* isolates characterized as insensitive exhibited from 667- to 1800-fold decreased *in vitro* sensitivity to pyraclostrobin when compared to the baseline isolates. Although the majority of isolates tested remained sensitive to pyraclostrobin, the development of QoI reduced-sensitive population in the prairie provinces in Canada was concerning for field pea producers in major field pea production regions of the US. From 2014 to 2016, 131 A*. pisi* isolates collected in Montana were determined to be sensitive to pyraclostrobin based on a discriminatory dose of 5 µg/ml (Owati et al., 2017). Reduced efficacy of QoI fungicides was first observed in *D. pinodes* in North Dakota in 2017; however, the distribution and frequency of insensitive isolates in the state has not been evaluated (Markell et al., 2018).

DMI fungicides are one of four classes of sterol biosynthesis inhibitors with broad-spectrum activity on many plant pathogens (Thomas et al., 2012). The mode of action of the DMI fungicides is the inhibition of fungal cell membrane development by interfering with ergosterol biosynthesis. Interference with this mechanism leads to disruption of membrane function, leakage of cytoplasmic contents, and hyphal death (Brent and Hollomon, 2007). Insensitivity to DMI fungicides has been reported in several phytopathogenic fungi since the 1980s (Fletcher and Wolfe, 1981; Schepers, 1983). Since that time, field resistance to DMI fungicides has developed in over 30 phytopathogenic fungi (Sheridan et al., 1985; Elad, 1992; Karaoglanidis et al., 2000; Reimann and Deising, 2005; Spolti et al., 2014). Despite their site-specific mode of action, DMI fungicides are considered to be at medium risk for fungicide resistance development FRAC Code List (2022). The development of insensitivity to DMI fungicides is a multi-step process, where decreases in disease control occur gradually (Brent and Hollomon, 2007). Prothioconazole (BayerCropScience, St. Louis, MO, USA) was first registered for use on field pea in 2007, and later, in combination with trifloxystrobin as Delaro^®^ SC (Bayer CropScience). Registration labels for both fungicides include limits of no more than three applications per season (Friskop et al., 2022).

There have been few reports assessing *in vitro* fungicide sensitivity of Ascochyta blight pathogens of field pea to prothioconazole. *In vitro* prothioconazole EC_50_ values ranged from 0.70 to 9.34 µg/ml for 52 baseline *D. pinodes* isolates collected from the US and prairie provinces of Canada (Delgado et al., 2011). *In vitro* sensitivity of a 2012 *D. pinodes* population from Montana was generally lower (EC_50 =_ 0.25 to 0.83 µg/ml) than was observed for the previously determined baseline population (Lonergan et al., 2015). A single *D. pinodes* isolate was reported with reduced-sensitivity *in vitro* to prothioconazole in a study conducted in China; however, only six isolates were evaluated (Liu et al., 2016). DMI fungicide sensitivity in *D. pinodes* and *A. pisi* pathogen populations from North Dakota have not been evaluated.

The objectives of this study were to determine: 1) the causal Ascochyta blight foliar pathogens of field pea present in North Dakota and their aggressiveness, 2) the *in vitro* sensitivity of *D. pinodes* and *A. pisi* populations from across field pea growing regions of North Dakota to pyraclostrobin and prothioconazole fungicides, and 3) the effect of any *in vitro* sensitivity shift on disease control.

## 2 Materials and methods

### 2.1 Pathogen collection, identification and maintenance

Isolates collected from 2017 to 2020 were obtained from infected leaf samples collected from field pea production fields in North Dakota and research plots located at North Dakota State University (NDSU) Research Extension Centers in Carrington, Langdon, Minot, and Williston (**Supplementary Tables S1, S2**). Symptomatic field pea leaves and stems containing lesions typical of Ascochyta blight were examined under a dissecting microscope for the presence of pycnidia. Tissue sections with lesions were surface sterilized in 1% NaOCl for 30 s, rinsed three times with sterile deionized water (SDW), and allowed to dry for 10 min in a laminar flow hood (Bowness et al., 2016). Tissue sections were aseptically transferred onto full-strength potato dextrose agar (PDA) (Difco™ Becton, Dickinson, and Company, Sparks, MD, USA) amended with streptomycin and neomycin (50 mg per milliliter). Cultures were incubated at 20 ± 2°C for 10 days under a cycle of alternating 12 h of white fluorescent light and 12 h of darkness (Onfroy et al., 1999). Individual isolates were differentiated morphologically by comparing conidial size, number of conidial cells, and macroscopic appearance of colony growth on media (Peever et al., 2007; Skoglund et al., 2011). Cultures generated from a single conidium transferred to fresh PDA and incubated for 14 days under the conditions previously described were considered distinct isolates. For working cultures (up to 6 months), 2.5-mm agar plugs bearing pycnidia were excised using a sterilized cork-borer, placed in a 1.5-ml sterile centrifuge tube with 1 ml of SDW, and stored at 4°C. The identity of the isolates collected was confirmed by PCR amplification and sequencing part of the rDNA internal transcribed spacer (ITS) region using primers ITS1 and ITS4 (Golani et al., 2016). Each isolates confirmed as acausal pathogen of Ascochyta blight was preserved for long-term cryogenic storage following previously described methods (Wise et al., 2008). Briefly, 2 µl of a conidial suspension was pipetted onto Whatman no. 1 filter paper that had been cut into small strips, autoclaved, and aseptically placed on the surface of PDA. After incubating for 14 days under conditions previously described, filter paper sections colonized by the fungus were aseptically removed, dried overnight in a laminar flow hood, placed in sterile 15-ml centrifuge tubes capped and sealed with Parafilm, and preserved at -80°C. Herbarium specimens were prepared for each plant sample from which *D. pinodes* and *A. pisi* isolates were obtained. Reference isolates were obtained for use in aggressiveness and fungicide sensitivity assays. Three *D. pinodes* isolates (Ap-1, Ap-8, and Mp-1) collected in 2001 were recovered from long-term cryogenic storage (**Supplementary Table S1**; Delgado et al., 2011). Two *A. pisi* isolates (D2 and Dp-3) previously tested to be sensitive to QoI fungicides were generously provided by Dr. Mary Burrows at Montana State University, Bozeman, MT, USA (**Supplementary Table S2**; Owati et al., 2017).

### 2.2 *In vitro* sensitivity of *D. pinodes* and *A. pisi* isolates to pyraclostrobin and prothioconazole

Pyraclostrobin and prothioconazole sensitivity was determined via mycelial growth assays conducted as described previously (Wise et al., 2011; Bowness et al., 2016). Working cultures were transferred onto PDA and were incubated under 24 h fluorescent light at 20 ± 2°C. After seven days, 5 mm agar plugs were excised from the leading edge of growth and inverted onto 100 × 15 mm Petri plates containing quarter-strength PDA (1/4 PDA) amended with technical grade formulations of pyraclostrobin (95% active ingredient [a.i.]) (BASF Corporation, Research Triangle Park, NC, USA). The fungicide was dissolved in acetone to reach final concentrations of 0, 0.01, 0.1, 1, 10, and 100 mg/ml (Bowness et al., 2016). Salicylhydroxamic acid (SHAM) (Sigma Chemical Company, St. Louis, MO), which has been previously determined to inhibit the alternative respiratory pathway, was dissolved in methanol at 100 µg/ml. The final concentration of both acetone and methanol in the media was 0.1% by volume. Technical grade prothioconazole (98.4% a.i.) (Bayer CropScience, Raleigh, NC, USA) was dissolved in acetone to reach final concentrations of 0, 0.01, 0.1, 1, 10, and 100 mg/ml. All amendments were added to the autoclaved media after it had cooled to 55°C.

Three baseline *D. pinodes* and two known sensitive *A. pisi* isolates were evaluated for *in vitro* sensitivity to both fungicides. The *D. pinodes* isolates were collected in 2001 and have no exposure to pyraclostrobin or prothioconazole. The *A. pisi* isolates were tested in previous research and were determined to be sensitive to pyraclostrobin (Owati et al., 2017). *D. pinode*s isolates collected from North Dakota in 2017 (11 isolates), 2018 (11 isolates), 2019 (69 isolates), and 2020 (44 isolates) were tested for *in vitro* sensitivity to both fungicides (**Table 1**; **Supplementary Table S1**). Additionally, *A. pisi* isolates collected in 2019 (44 isolates) and 2020 (six isolates) were tested for *in vitro* sensitivity to both fungicides (**Table 1**; **Supplementary T** **able S2**). *D. pinodes* control isolates Ap-1, Ap-8, and Mp-1 and *A. pisi* control isolates D-1 and DP-1 were included in *in vitro* assays as internal controls to determine assay reproducibility for each pathogen (Wong and Wilcox, 2002). Two perpendicular measurements of mycelial growth for each isolate at all fungicide concentrations were recorded, with the original plug diameter (5 mm) subtracted, after incubation at 20 ± 2°C in darkness for 14 days (Bowness et al., 2016).

**Table 1:** Collection information for *Didymella pinodes* and *Ascochyta pisi* isolates collected in North Dakota and Montana from 2001 to 2020.

Year	State/county of origin	No. of *D. pinodes* isolates	No. of *A. pisi* isolates
2001^a^	North Dakota	3	–
2015^b^	Montana	–	1
2016^b^	Montana	–	1
2017	Cavalier	9	–
	Foster	6	–
2018	Foster	28	–
2019	Burke	1	2
	Burleigh	4	1
	Cass	12	4
	Cavalier	8	4
	Foster	15	14
	McHenry	1	2
	McKenzie	2	3
	McLean	5	7
	Mountrail	17	8
	Rolette	9	3
	Walsh	8	5
	Ward	5	4
	Wells	3	2
2020	Burke	5	1
	Cavalier	24	3
	Foster	6	1
	Mountrail	4	–
	Ward	3	1
	Williams	2	-

a *Didymella pinodes* isolates collected from North Dakota in 2001 had no exposure to pyraclostrobin and prothioconazole and, hence, are categorized as baseline isolates (Delgado et al., 2011)

b *Ascochyta pisi* isolates collected from Montana in 2015 and 2016 were determined to be sensitive to pyraclostrobin in previous studies (Owati et al., 2017).

### 2.3 Aggressiveness of *D. pinodes* and *A. pisi* isolates

**Figure 2:**
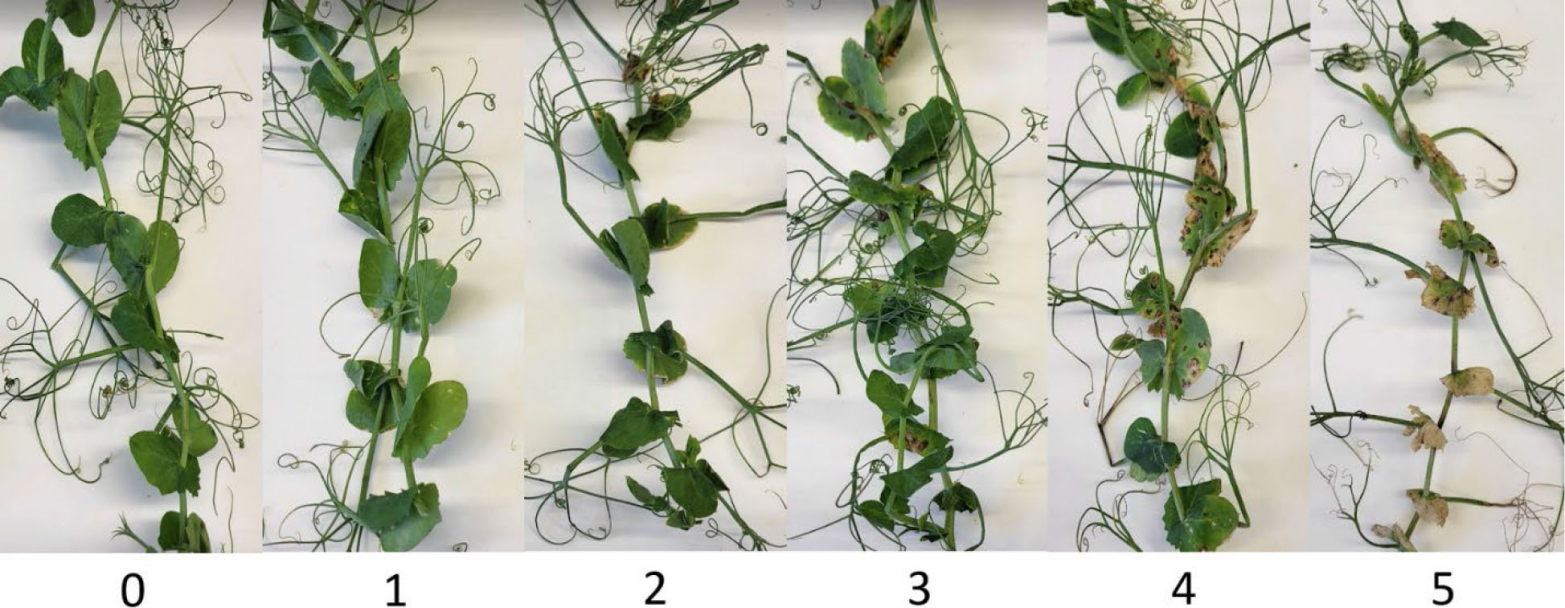
Ascochyta blight severity rating scale used to evaluate disease in the greenhouse; 0 = no disease; 1 = a few dispersed lesions; 2 = several lesions; 3 = 10 to 15% of leaf area necrotic; 4 = 50% of the leaf area dehydrated and/or covered by lesions; 5 = 75 to 100% of the leaf area dehydrated and/or necrotic (Adapted from Onfroy et al., 1999).

To determine if *D. pinodes* and *A. pisi* isolates were pathogenic to field peas and estimate aggressiveness, trials on a subset of isolates were conducted under greenhouse conditions in the NDSU Agriculture Experiment Station Jack Dalrymple Research Complex. Selected *D. pinodes* isolates included four collected in 2017, two collected in 2018 and two collected in 2001 known to be sensitive to both fungicides (used as baseline). Selected *A. pisi* isolates included four collected in 2019 and two known to be sensitive to both fungicides (collected in 2015 and 2016) (**Table 2**). Three seeds of an Ascochyta blight susceptible field pea cultivar ‘DS Admiral’ (Andersen et al., 2002) were sown in each 15.2 × 8.9 × 8.9 cm pot containing Sunshine Mix LC1 (Sun Gro Horticulture Inc., Bellevue, WA, USA). After emergence, plants were thinned to obtain one uniformly sized plant per pot and maintained at 25 ± 2°C with daily application of water. After three weeks, plants were inoculated with a conidial suspension produced from four- to seven-day-old-cultures of a single isolate of *D. pinodes* or *A. pisi* maintained on pea agar (Field pea cultivar ‘DS Admiral’, 50 g; agar, 15 g; and distilled water, 1000 ml) under 24 h fluorescent light at 22 ± 2°C. Conidia were harvested by adding 0.01% Tween 20 (100 µl Tween 20 in 1000 ml distilled water) and scraping the agar surface with a cotton swab. The suspension was filtered through a double layer of cheesecloth and adjusted to 1 × 10^5^ conidia/ml using a hemocytometer. The conidial suspension was applied to the plants to run-off using a paint-spray gun (Anest Iwata-Medea Inc, Portland, OR, USA). To avoid cross-infection among isolates, inoculated plants were incubated in individual humidity chambers (Phytotronic Inc., Earth City, MO, USA) for 48 h at >95% relativity humidity and 22 ± 2°C and transferred to confinement chambers (plastic chambers with open ceiling) on greenhouse benches. Ascochyta blight severity was rated visually at 14 days post inoculation (DPI) on the four lowest leaves (four sub-samples) based on a visual scale of 0 to 5: 0 = no disease; 1 = a few dispersed lesions; 2 = several lesions; 3 = 10 to 15% of leaf area necrotic; 4 = 50% of the leaf areas dehydrated and/or covered by lesions; 5 = 75 to 100% of the leaf area dehydrated and/or necrotic (**Figure 2**; adapted from Onfroy et al., 1999).

**Table 2:** Aggressiveness, *in vitro* and *in vivo* fungicide sensitivities for *Didymella pinodes* and *Ascochyta pisi* isolates.

Isolate		State/county of origin	Collection year	Disease severity (%)^c^		Pyraclostrobin			Prothioconazole		
						EC_50_ (µg/ml)^d^	AUDRC^e^		EC_50_ (µg/ml)	AUDRC	
				Mean	Std dev		Mean	Std dev		Mean	Std dev
D. pinodes											
Ap-1^a^		North Dakota	2001	78.2 a	16.7	0.04	7740.9 a	786.2	0.1	9498.6 a	415.3
Ap-8^a^		North Dakota	2001	78.2 a	16.7	0.05	8199.9 a	534.4	0.32	9279.9 a	509.7
T2R5-1		ND/Foster	2018	83.8 a	11.5	0.18	5533.2 c	664.4	1.84	8076.6 c	512.0
T1R2-6		ND/Foster	2018	80.1 a	15.4	0.27	6405.3 b	468.5	12.33	7214.4 d	548.3
2-3		ND/Cavalier	2017	80.0 a	15.4	13.43	4557.6 d	648.7	0.83	8760.6 b	315.0
C2		ND/Foster	2017	85.6 a	8.4	35.71	1836.0 e	631.0	51.17	7212.6 d	546.7
3-3		ND/Cavalier	2017	80.1 a	13.7	100	4240.8 d	508.1	1.01	8861.4 b	417.0
C1		ND/Foster	2017	85.6 a	8.4	100	2251.8 e	783.9	6.06	7196.4 d	508.2
LSD*_P_*_=0.05_ ^f^				NS			743.5			411.7	
A. pisi											
Dp-3^b^		Montana	2016	56.1 c	25.2	0.35	8825.4 a	295.9	0.15	9081.9 a	517.8
D2^b^		Montana	2015	57.9 bc	23.1	0.61	9298.8 a	503.8	0.61	9300.6 a	505.2
17-1		ND/McLean	2019	74.4 a	18.4	1.44	6732.0 b	635.7	5.01	7196.4 c	530.8
10-4		ND/Mountrail	2019	69.0 ab	19.2	4.15	4680.0 c	773.4	0.97	8841.6 a	427.1
4-2		ND/Rolette	2019	83.8 a	11.5	100	4401.9 d	489.7	4.13	8037.9 b	499.1
30-5		ND/Foster	2019	78.3 a	16.7	100	2688.3 d	759.6	3.02	7255.8 c	514.5
LSD*_P_*_=0.05_				12.5			532.1			487.1	

a Baseline isolates, collected prior to fungicide registration (Delgado et al., 2011).

b Determined to be sensitive to pyraclostrobin in previous studies (Owati et al., 2017).

c Aggressiveness data were transformed from a 0 to 5 scale to percent disease severity (Onfroy et al., 1999).

d Effective concentration at which the fungal growth is inhibited by 50% (EC_50_) were obtained for both fungicides from the in vitro assessments.

e Area under the dose response curve (AUDRC) generated under greenhouse conditions.

f Means followed by same letters are not significantly different based Fisher’s protected least significant difference (α = 0.05).

### 2.4 *In vivo* efficacy of pyraclostrobin and prothioconazole on *D. pinodes* and *A. pisi*

The impact of an *in vitro* shift in sensitivity of *D. pinodes* and *A. pisi* to pyraclostrobin and prothioconazole on disease control was determined under greenhouse conditions using the same subset of *D. pinodes* and *A. pisi* isolates evaluated for pathogenicity and aggressiveness. The *in vivo* fungicide efficacy assays were conducted as a 24-h preventive test (Fonseka and Gudmestad, 2016). Pea plants, reared as described above for pathogenicity and aggressiveness trials, were treated with a commercial formulation of pyraclostrobin (23.3% a.i.) (Headline ^®^ SC, BASF Corporation) or prothioconazole (41% a.i.) (Proline ^®^ SC, Bayer CropScience). Ten-fold fungicide dilutions (0, 0.1, 1, 10, and 100 µg active ingredient/ml) were applied to plants to obtain a dose response curve. Fungicide was applied using a Generation II Research Sprayer (Devries Manufacturing, Hollandale, MN, USA) at approximately 400 kPa and plants were returned to the greenhouse room. Twenty-four hours after fungicide application, plants were inoculated with a *D. pinodes* or *A. pisi* conidial suspension and disease severity was assessed as described above for aggressiveness assays.

### 2.5 Statistical analyses

All *in vitro* experiments for each pathogen × fungicide combination were performed twice in a completely random design with two replications (plates) at each fungicide concentration. EC_50_ values were calculated for each isolate using the percentage reduction in mycelial growth relative to the non-fungicide amended control (Fonseka and Gudmestad, 2016). Data were regressed against the log_10_ fungicide concentration and the EC_50_ value was determined by interpolation of 50% intercept using the Statistical Analysis System (SAS^©^ Institute Inc., Cary, NC, USA). EC_50_ values of <0.01 and >100 were considered as 0.01 and 100 µg/ml, respectively, for final analysis (Budde-Rodriguez et al., 2021). The assay reproducibility calculations generated approximate limits for 95% confidence intervals for the internal controls in every trial (Wong and Wilcox, 2002). Within pathogens and fungicides, the F-test was used to determine homogeneity of variance across *in vitro* experiments and Fisher’s protected least significant difference (LSD) test was used for mean separation of EC_50_ values across years isolates were collected (α = 0.05). A resistance factor (Rf) was calculated by dividing the EC_50_ value of each isolate collected from 2017 to 2020 by the mean EC_50_ value of the baseline/known sensitive isolates for each pathogen and fungicide (Brent and Hollomon, 2007).

All greenhouse experiments were conducted twice in split-plot randomized complete block designs with *D. pinodes* or *A. pisi* isolates as the main plot and fungicide concentration as split-plots. Five replications (five pots) were tested for each isolate × fungicide concentration. Ordinal rating data were converted to percent disease control by each pathogen isolate × fungicide combination using the formula [1 – (Disease rating/disease rating in non-treated plants) × 100]. Homogeneity of variance among two independent trials was determined via Levene’s test (Milliken and Johnson, 1992). Data from both trials were combined, and “trial” was considered as a random effect. Data from aggressiveness assays were treated as randomized complete blocks nested within fungicide where both “block” and “fungicide” were considered random effects in the model. Analysis of variance was implemented with the Glimmix procedure in SAS version 9.4. The area under the dose-response curve (AUDRC) was calculated to determine significant differences in disease control provided by pyraclostrobin or prothioconazole (Fonseka and Gudmestad, 2016). Fisher’s protected least significant difference (LSD; α = 0.05) test was used for the mean separation of isolate aggressiveness within each pathogen and AUDRC within each pathogen and fungicide. Regression models were implemented within fungicides to elucidate the relationship between disease control and *in vitro* sensitivity of the isolates used in the greenhouse trials. AUDRC was modeled with a linear-log simple regression to EC_50_. Models were fitted with the “lm” function in R (R Core Team, 2022), and figures were produced with the package ggplot2 (Wickham, 2016).

## 3 Results

### 3.1 Pathogen identification

**Figure 3:**
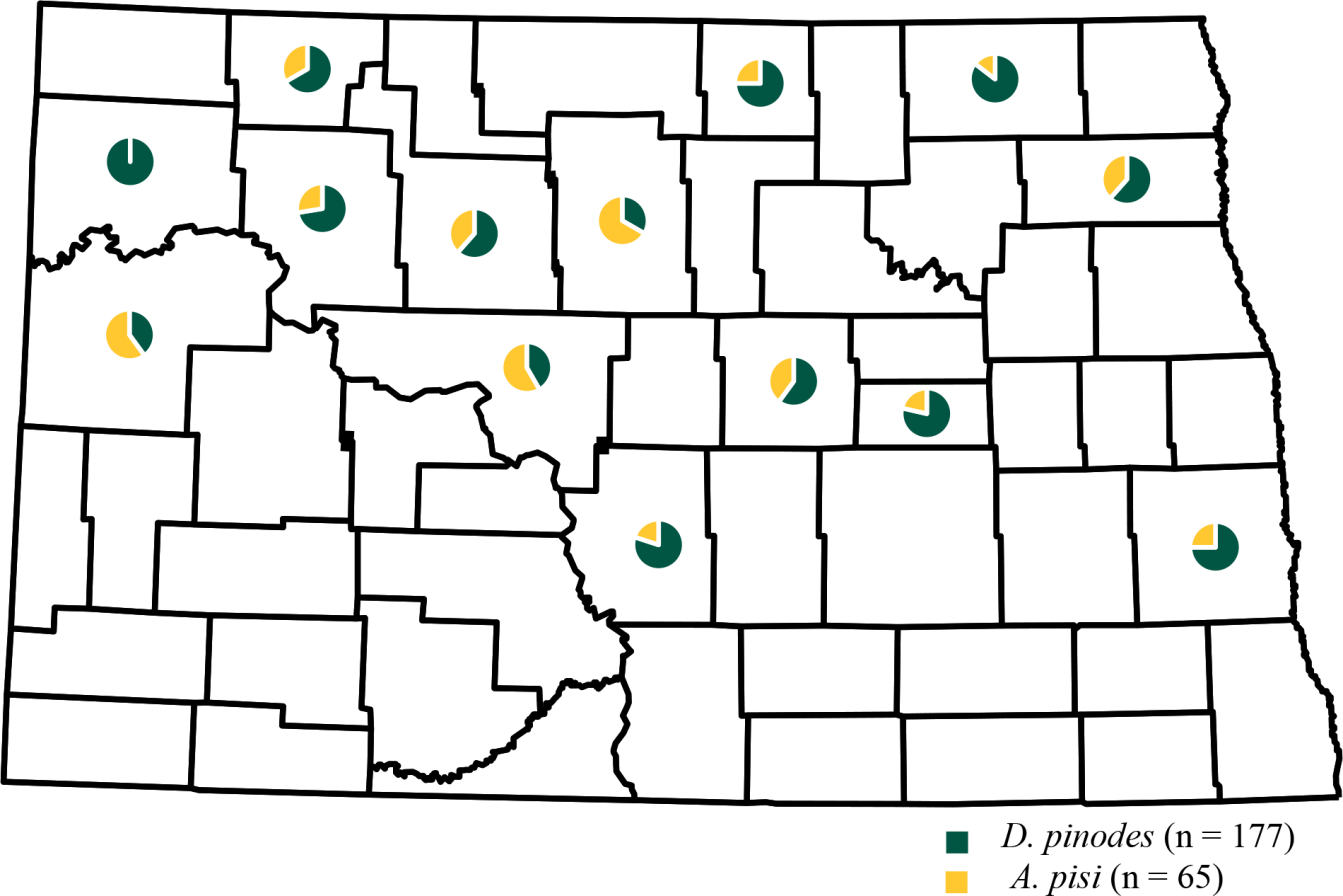
Geographic frequency distribution for *Didymella pinodes* collected from 2017 to 2020 and *Ascochyta pisi* isolates collected from 2019 to 2020 in North Dakota.

Leaf samples with characteristic Ascochyta blight lesions were obtained from 56 field pea production fields in 14 counties in North Dakota from 2017 to 2020 (**Table 1**; **Figure 3**). *D. pinodes* was recovered most frequently (73%; n = 177) and from all years and all counties surveyed. *A. pisi* (27%; n = 65) and was recovered in 2019 and 2020 from all counties surveyed with the exception of Williams county in northwestern North Dakota. Although *D. pinodes* was the predominant pathogen isolated from the counties surveyed in 2019 and 2020, *A. pisi* was the dominant species isolated from McHenry (67%), McKenzie (60%), and McLean (58%) counties.

### 3.2 *In vitro* sensitivity of *D. pinodes* and *A. pisi* isolates to pyraclostrobin and prothioconazole

**Figure 4:**
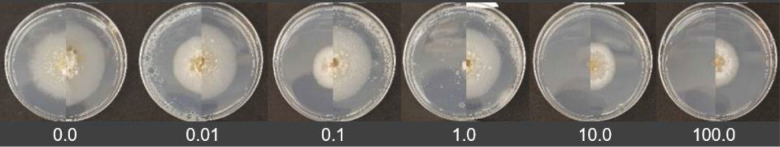
*In vitro* assays comparing a baseline (left) and a 2017 to 2020 (right) *Didymella pinodes* isolate to pyraclostrobin. The fungicide concentration increases from 0.0 ppm (left) to 100 ppm (right).

**Figure 5:**
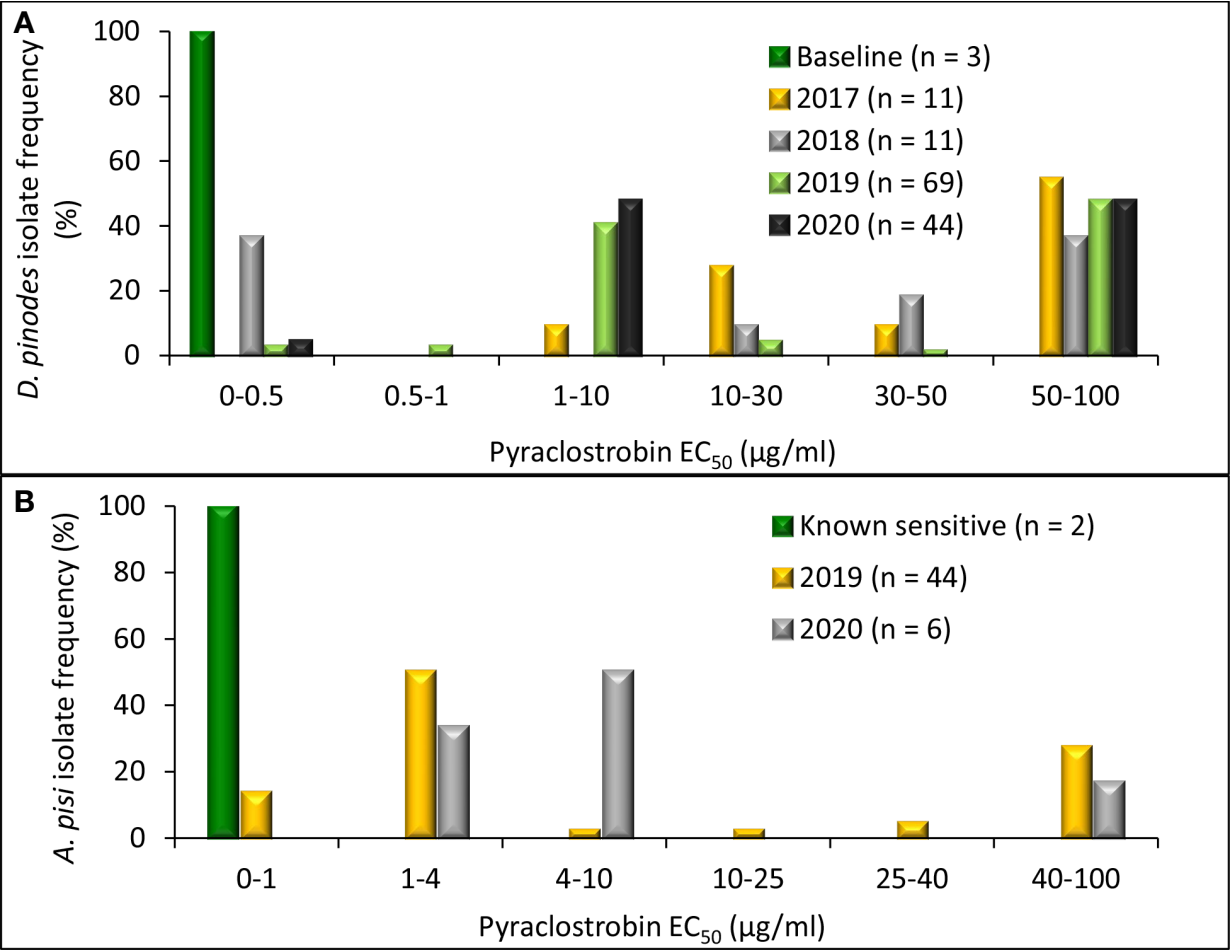
Frequency distribution of *in vitro* sensitivity to pyraclostrobin based on methods to determine the effective concentration that inhibits mycelial growth by 50% when compared with the non-fungicide-amended (EC_50_ µg/ml) for **(A)** *Didymella pinodes* (n = 138) isolates collected from 2001 (baseline; Delgado et al., 2011) and 2017 to 2020 and **(B)**, *Ascochyta pisi* (n = 52) isolates collected from 2015 and 2016 (determined to be sensitive to QoI fungicides; Owati et al., 2017), and isolates collected in 2019 and 2020).

Radial growth assays uncovered an *in vitro* shift in fungicide sensitivity among isolates evaluated (**Figure 4**). Variances were homogenous for *D. pinodes* (*P* = 0.6095) and *A. pisi* (*P* = 0.2755); thus, trials were combined within pathogens. EC_50_ values of the three baseline *D. pinodes* isolates ranged from 0.04 to 0.12 µg/ml for pyraclostrobin (**Figure 5A**; **Supplementary T** **able S1**), within the previously established baseline of 0.03 to 0.29 µg/ml (Bowness et al., 2016). EC_50_ values of *D. pinodes* isolates collected from 2017 to 2020 ranged from 0.17 to >100 µg/ml. *In vitro* sensitivity of 100% (n = 135) of isolates collected from 2017 to 2020 was lower than the sensitivity of the three baseline isolates evaluated during the current study. When compared to the previously established baseline (0.03 to 0.29 µg/ml; Bowness et al., 2016), 96% (129) of *D. pinodes* isolates were less sensitive to pyraclostrobin. Thirty-six percent (n = 49) of isolates displayed EC_50_ values >100 µg/ml. Rf for *D. pinodes in vitro* pyraclostrobin sensitivity ranged from 2.4 to 1,429. EC_50_ values of the two known sensitive *A. pisi* isolates included in this study were 0.35 (Dp-3) and 0.61 µg/ml (D2) for pyraclostrobin (**Figure 5B**; **Supplementary Table S2**). EC_50_ values of *A. pisi* isolates collected from 2019 to 2020 ranged from 0.56 to 100 µg/ml. *In vitro* sensitivity of 98% (n = 49) of *A. pisi* isolates collected from 2019 to 2020 were lower than the sensitivity of the two known sensitive isolates tested during this evaluation. Eighteen percent of *A. pisi* isolates displayed EC_50_ values >100 µg/ml pyraclostrobin. Rf for *A. pisi in vitro* sensitivity ranged from 1.2 to 208.3.

**Figure 6:**
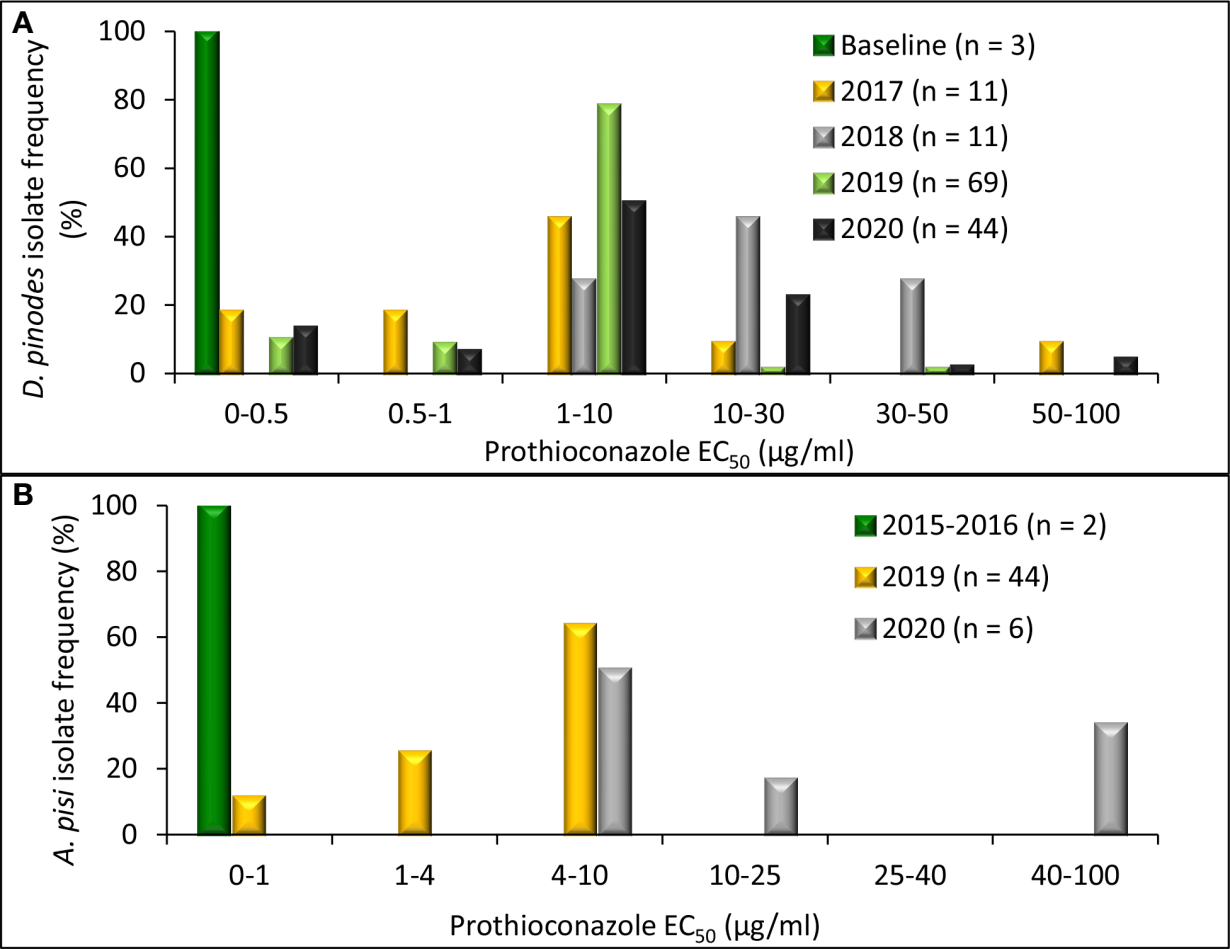
Frequency distribution of *in vitro* sensitivity to prothioconazole based on methods to determine the effective concentration that inhibits mycelial growth by 50% when compared with the non-fungicide-amended (EC_50_ µg/ml) for **(A)**, *Didymella pinodes* (n = 138) isolates collected from 2001 (baseline; Delgado et al., 2011) and 2017 to 2020 and **(B)**, *Ascochyta pisi* (n = 52) isolates collected from 2015 and 2016 (Owati et al., 2017), and isolates collected in 2019 and 2020).

Independent ANOVA of *in vitro* prothioconazole sensitivity assays determined that variances were homogenous for *D. pinodes* (*P* = 0.1690) and *A. pisi* (*P* = 0.8356); thus, trials were combined within pathogens. EC_50_ values of the three baseline *D. pinodes* isolates ranged from 0.10 to 0.32 µg/ml for prothioconazole (**Figure 6A**; **Supplementary Table S1**). This is below the range of the previously established baseline of 0.70 to 9.34 µg/ml (Delgado et al., 2011) and below or within the 2012 Montana population of 0.25 to 0.83 µg/ml (Lonergan et al., 2015). EC_50_ values of *D. pinodes* isolates collected from 2017 to 2020 ranged from 0.10 to 67.6 µg/ml for prothioconazole. *In vitro* prothioconazole sensitivities of 91% (n = 123) of isolates collected from 2017 to 2020 were lower than the sensitivities of the three baseline *D. pinodes* isolates evaluated during the current study. Nineteen percent (26 isolates) had lower sensitivity than the previously established prothioconazole baseline (0.70 to 9.34 µg/ml; Delgado et al., 2011) and 83% (112 isolates) displayed lower sensitivity than the 2012 Montana population (0.25 to 0.83 µg/ml; Lonergan et al., 2015). Rf for *D. pinodes in vitro* pyraclostrobin sensitivity ranged from 0.5 to 337.9. EC_50_ values of the two known sensitive *A. pisi* isolates included in this study were 0.15 (Dp-3) and 0.61 µg/ml (D2) for prothioconazole (**Figure 6B**; **Supplementary Table S2**). EC_50_ values of *A. pisi* isolates collected from 2019 to 2020 ranged from 0.34 to 48.3 µg/ml. *In vitro* prothioconazole sensitivity of 98% (n = 49) of *A. pisi* isolates collected in 2019 and 2020 were lower than the two known sensitive *A. pisi* isolates tested during this evaluation. Rf for *A. pisi in vitro* sensitivity ranged from 0.9 to 127.0 for prothioconazole.

**Figure 7:**
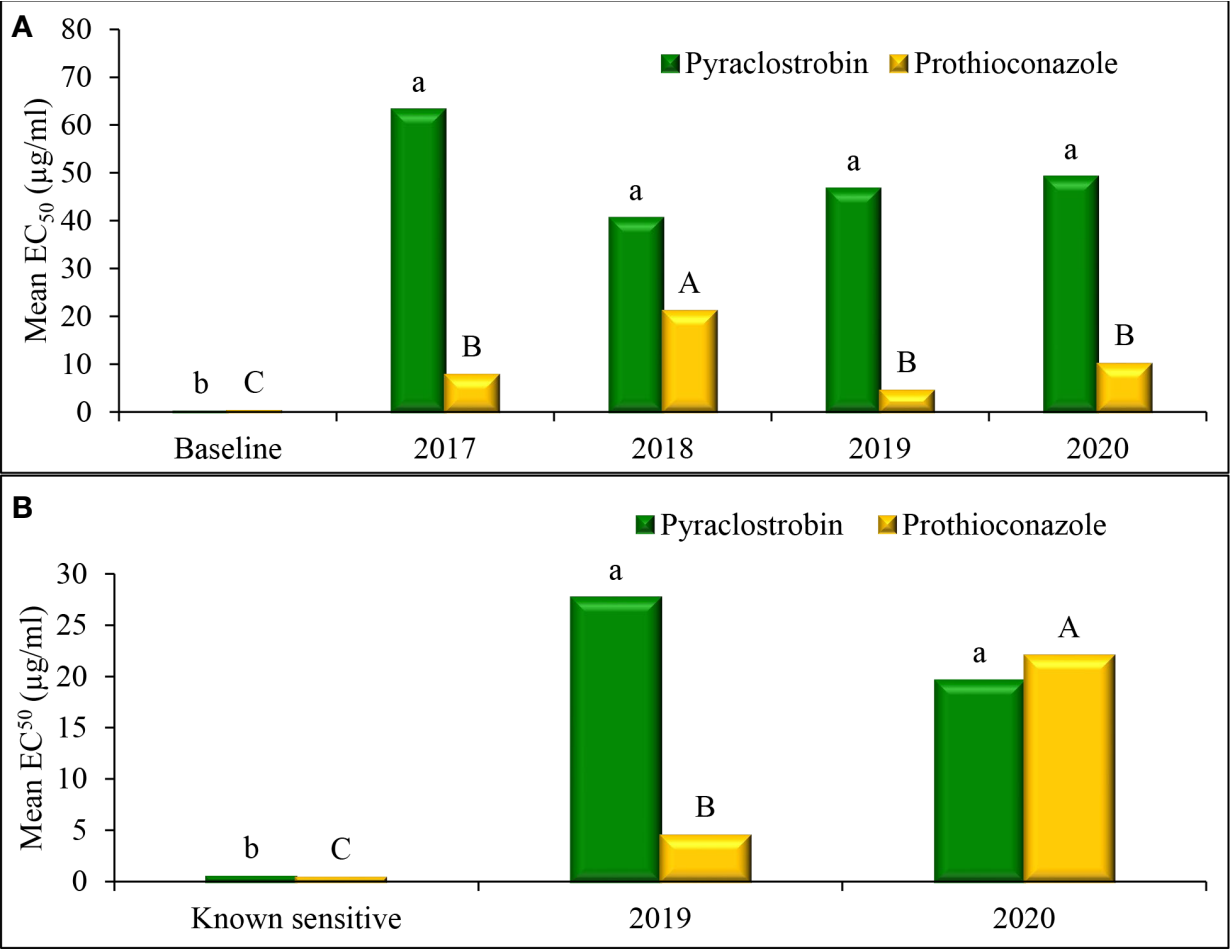
Mean effective concentration that inhibits mycelial growth by 50% compared with the non-fungicide-amended (EC_50_ µg/ml) for *in vitro* isolate sensitivity of **(A)** *Didymella pinodes* (n = 138) and **(B)** *Ascochyta pi*s*i* (n = 52), to pyraclostrobin and prothioconazole across years. Within species, columns with the same letter are not significantly different based on Fisher’s protected least significant difference (α = 0.05).

Mean EC_50_ values of baseline *D. pinodes* isolates was 0.07 µg/ml for pyraclostrobin and was 0.20 µg/ml for prothioconazole (**Figure 7A**). Mean sensitivities of isolates collected from 2017 to 2020 were significantly lower than the mean of the baseline isolates for both fungicides. No significant difference in sensitivity was observed across isolates within years for non-baseline populations for pyraclostrobin. Mean prothioconazole sensitivity of *D. pinodes* isolates collected in 2018 was significantly lower when compared to isolates collected in 2017, 2019, and 2020. Mean EC_50_ values of known sensitive *A. pisi* isolates were 0.48 µg/ml for pyraclostrobin and 0.38 µg/ml for prothioconazole (**Figure 7B**). Mean sensitivities of isolates collected in 2019 to 2020 were significantly lower than the mean sensitivities of baseline isolates for both fungicides. *A. pisi* isolates collected in 2019 and 2020 did not differ in mean sensitivity to pyraclostrobin; however, isolates collected in 2020 displayed significantly lower mean sensitivity to prothioconazole when compared to isolates collected in 2019.

**Figure 8:**
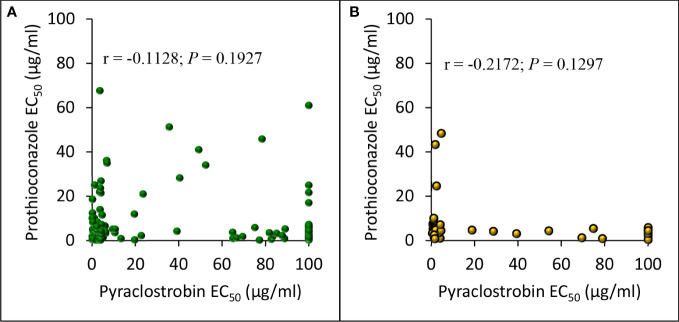
Pearson’s correlation between *in vitro* pyraclostrobin and prothioconazole sensitivity (EC_50_ µg/ml) of **(A)**, *Didymella pinodes* (n = 135) and **(B)**, *Ascochyta pisi* (n = 50) isolates collected from 2017 to 2020, and 2019 to 2020, respectively.

Pearson correlation analysis comparing EC_50_ values for pyraclostrobin and prothioconazole sensitivities of *D. pinodes* isolates collected from 2017 to 2020 was not significant (*r* = -0.1128; *P* = 0.1927); however, sensitivities of a sub-set of isolates appear to be correlated (**Figure 8A**). Pearson correlation analysis comparing EC_50_ values for pyraclostrobin and prothioconazole sensitivities of individual *A. pisi* isolates collected from 2019 to 2020 was not significant (*r* = -0.2172; *P* = 0.1297) (**Figure 8B**). However, *in vitro* sensitivities for both pyraclostrobin and prothioconazole of 92% (n = 124) of *D. pinodes* and 98% (n = 49) of *A. pisi* isolates collected during the disease survey from 2017 to 2020 were lower than the sensitivities of the baseline/known sensitive isolates evaluated during the current study (**Supplementary Table S1**; **Supplementary T** **able S2**).

### 3.3 Aggressiveness of *D. pinodes* and *A. pisi* isolates

All eight *D. pinodes* and six *A. pisi* isolates evaluated in the greenhouse were determined to be pathogenic to cultivar ‘DS Admiral’. Disease severity caused by isolates of *D. pinodes* ranged from 78 to 86% and was not significantly different across the eight isolates evaluated (**Table 2**). Significant differences were observed across the six *A. pisi* isolates evaluated, where disease severity ranged from 56 to nearly 84%. Isolate Dp-3, collected in 2016, was significantly less aggressive than all other *A. pisi* isolates with the exception of isolate D2, collected in Montana in 2015. Aggressiveness of *A. pisi* isolate D2 was not different than isolate 10-4 collected from North Dakota in 2019. No significant difference in aggressiveness was observed among the four *A. pisi* isolates collected from North Dakota in 2019.

### 3.4 *In vivo* efficacy of pyraclostrobin and prothioconazole on *D. pinodes* and *A. pisi*

**Figure 9:**
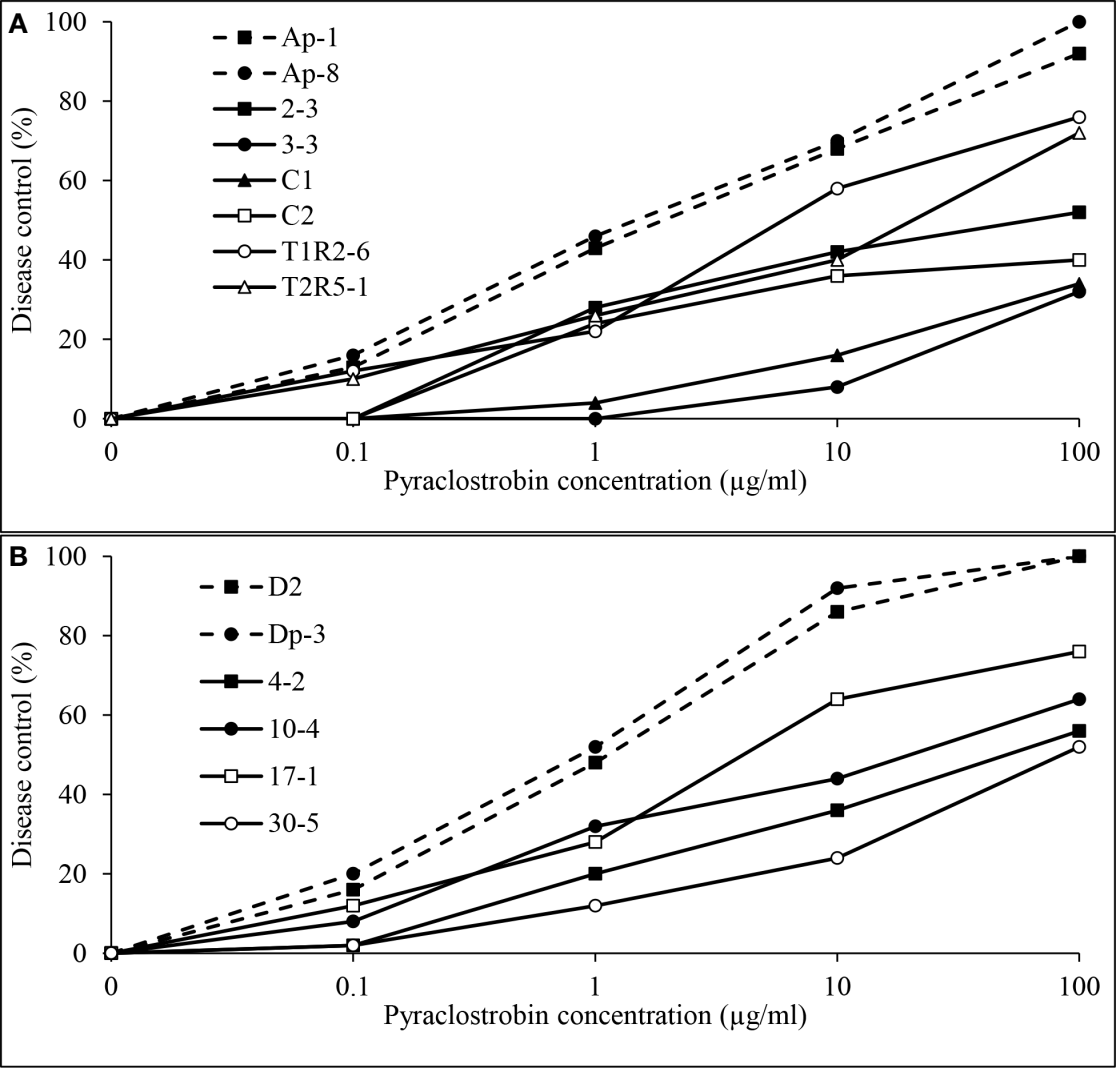
Mean *in vivo* percentage disease control by pyraclostrobin of **(A)**, two baseline (Ap-1 and Ap-8; Delgado et al., 2011) and six *Didymella pinodes* isolates collected in 2017 and 2018 and **(B)**, two *Ascochyta pisi* isolates collected from 2015 and 2016 (D2 and Dp-3 determined to be sensitive to pyraclostrobin; Owati et al., 2017) and four isolates collected in 2019 as determined in greenhouse assays.

Independent analysis of pyraclostrobin *in vivo* disease control experiments for *D. pinodes* (*P* = 0.1086) and *A. pisi* (*P* = 0.6314) determined that variances were homogenous; thus, trials were combined within pathogen for further analysis. Significant differences in disease control provided by pyraclostrobin were observed among *D. pinodes* isolates (**Table 2**; **Figure 9A**). Statistical analyses divided isolates into five groups based on AUDRC, with the two baseline isolates controlled at a significantly higher level than the six non-baseline isolates evaluated. Non-baseline isolates were generally broken down by 10-fold decreases in *in vitro* sensitivity. As *in vitro* sensitivity decreased (increasing EC_50_ value), control provided by the fungicide decreased (decreasing AUDRC). Disease control provided by pyraclostrobin of the four isolates of *A. pisi* collected in 2019 was significantly lower than that of known sensitive isolates D2 and Dp-3 (**Table 2**; **Figure 9B**). *A. pisi* isolates collected in 2019 were divided into three statistical groups following trends in *in vitro* sensitivity.

**Figure 10:**
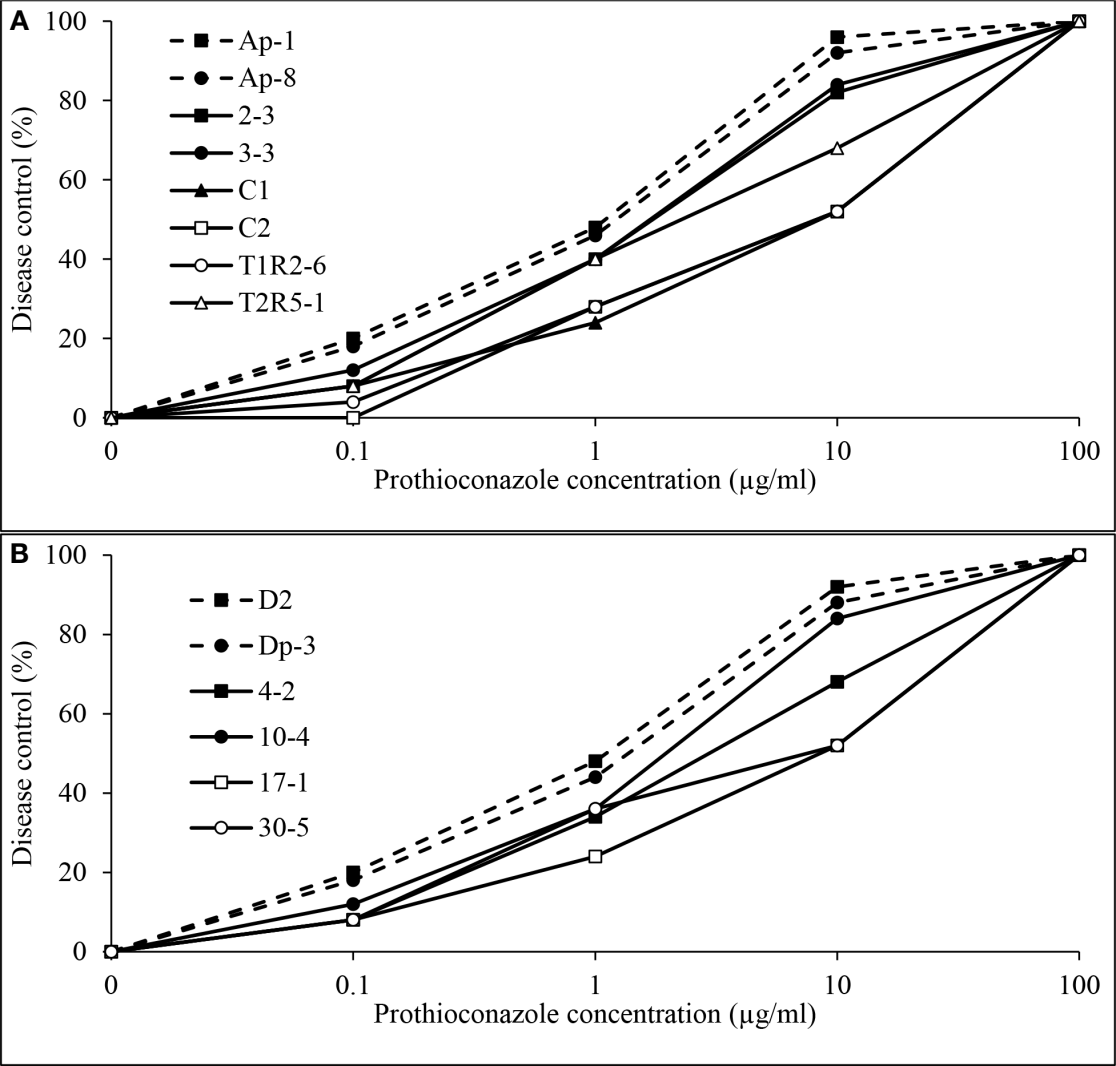
Mean *in vivo* percentage disease control by prothioconazole of **(A)**, two baseline (Ap-1 and Ap-8; Delgado et al., 2011) and six *Didymella pinodes* isolates collected in 2017 and 2018 and **(B)**, two *Ascochyta pisi* isolates collected from 2015 and 2016 (D2 and Dp-3; Owati et al., 2017) and four isolates collected in 2019 as determined in greenhouse assays.

**Figure 11:**
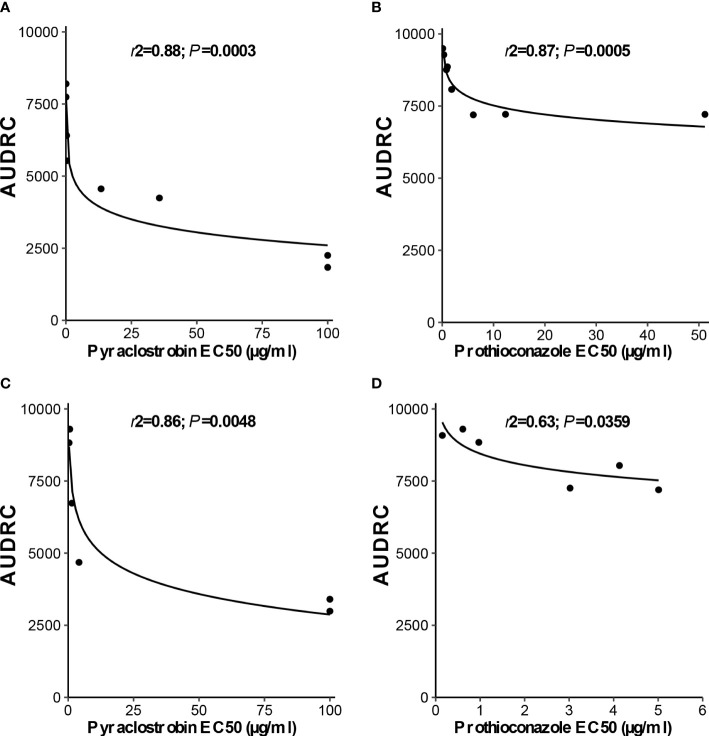
Linear-log simple regression comparing *in vitro* **(A, C)**, pyraclostrobin and **(B, D)**, prothioconazole sensitivity (EC_50_ µg/ml) and area under dose response curve values for **(A, B)**, *Didymella pinodes* and **(C, D)**, *Ascochyta pisi* isolates.

Independent analysis of prothioconazole *in vivo* disease control experiments for *D. pinodes* (*P* = 0.7465) and *A. pisi* (*P* = 0.5217) determined that variances were homogenous and repetitions were combined within pathogen for further analysis. Disease control provided by prothioconazole of non-baseline isolates of *D. pinodes* was significantly lower than control of baseline isolates (**Table 2**; **Figure 10A**). Control of non-baseline isolates was statistically classified into three groups. Similar to the observations with pyraclostrobin, with decreasing *in vitro* sensitivity, disease control provided by prothioconazole also decreased (**Table 2**). Disease control of three isolates of *A. pisi* collected in 2019 was significantly less than the disease control provided by prothioconazole on known sensitive isolates (**Table 2**; **Figure 10B**). One isolate, 10-4, displayed similar prothioconazole *in vitro* sensitivity to known sensitive *A. pisi* isolates and was controlled similarly to those isolates under greenhouse conditions (**Table 2**). Linear-log simple regression analysis disclosed a significant relationship (r^2^ from 0.63 to 0.88) between *in vitro* EC_50_ values and *in vivo* AUDRC for all four fungicide:pathogen combinations (**Figures 11A-D**).

## 4 Discussion

Results from this research demonstrate that *D. pinodes* is the dominant foliar Ascochyta blight pathogen in North Dakota, but that *A. pisi* coexists and plays an important role in the state, particularly in some regions. This study also established a robust survey and monitoring approach to examine pathogen sensitivity to fungicides, and reports a shift of sensitivity of *D. pinodes* and *A. pisi* to pyraclostrobin and prothioconazole in *in vitro* and greenhouse assays. Pathogen prevalence, aggressiveness and fungicide sensitivity presented in this study contribute crucial knowledge which helps determine research priorities and integrated pest management recommendations to growers in North Dakota and the region.

Three fungal pathogens are associated with the Ascochyta blight disease complex in North America (Aveskamp et al., 2010). *D. pinodella* has been associated with disease complex in field pea seed lots tested in Montana and Saskatchewan (Sivachandra Kumar and Banniza, 2017; Owati et al., 2020), and became the prevalent species in Canada in the mid-1960s after the introduction of host resistance to *A. pisi* (Tivoli and Baninza, 2007). However, this pathogen was not detected during the North Dakota survey. This is likely because we isolated only from foliar and stem lesions with symptoms characteristic of Ascochyta blight; *D. pinodella* has been frequently isolated from pea roots, which were not evaluated during the current study (Persson et al., 1997). Differences in environmental conditions, location, and cropping systems also have been reported to impact the ecological balance among species involved in the Ascochyta blight complex of field pea (Barbetti et al., 2021).

The current research determined that all *D. pinodes* and *A. pisi* isolates evaluated were pathogenic on cultivar ‘DS Admiral’, and no difference was observed in aggressiveness among eight isolates of *D. pinodes*. Aggressiveness of two of six *A. pisi* isolates fell within the range of the *D. pinodes* isolates, while two *A. pisi* isolates from Montana were significantly less aggressive than *A. pisi* isolates recently collected from North Dakota. These results are only somewhat consistent with previous research indicating higher aggressiveness of *D. pinodes* when compared to *A. pisi* (Owati et al., 2020). Collection and storage conditions may have contributed to observed differences.

This is the first report we are aware of monitoring sensitivity of *D. pinodes* and *A. pisi* populations to pyraclostrobin and prothioconazole across multiple years. Evaluating a large number of isolates from a wider geographic area is critical in fungicide sensitivity monitoring, especially when loss of disease control has not been detected (Russell, 2008). *In vitro* inhibition of mycelial growth is less resource intensive than evaluating spore germination and has been demonstrated effective in detecting shifts in pyraclostrobin sensitivity in *D. pinodes* (Bowness et al., 2016). This previous research demonstrated that despite the EC_50_ values of the baseline *D. pinodes* isolates being considerably lower using spore germination when compared to mycelial growth, no differences were observed in the classification of isolates as sensitive or resistant when comparing methods. During our evaluations, *in vitro* sensitivity of the three baseline *D. pinodes* isolates collected in 2001 from North Dakota fell within the previously established baseline established using the mycelial growth assay. *In vitro* sensitivity of the two *A. pisi* isolates obtained from Montana were previously determined to be sensitive to pyraclostrobin using a discriminatory dose and a mismatch amplification mutation assay to detect the G143A mutation; therefore, direct comparisons could not be made to the *in vitro* EC_50_ results generated in our study (Owati et al., 2017).

Reductions in the efficacy of pyraclostrobin for the management of field pea Ascochyta blight were first observed in North Dakota in 2017 (Markell et al., 2018). At that time, *D. pinodes* isolates were obtained from typical Ascochyta blight foliar lesions from two field sites where fungicide efficacy appeared uncharacteristically low. All 14 isolates displayed reduced-sensitivity/resistance to pyraclostrobin with Rf ranging from 89.0 to 1,428.6 and reductions in efficacy were confirmed under greenhouse conditions. These observations prompted us to initiate a widescale monitoring effort to obtain a thorough understanding of the frequency and distribution of fungicide reduced-sensitivity/resistance in Ascochyta blight pathogens. Among *D. pinodes* and *A. pisi* isolates collected from 2017 to 2020, pyraclostrobin resistance and/or reduced-sensitivity were ubiquitous across sampling sites in North Dakota. All isolates collected from 2017 to 2020 were less sensitive to pyraclostrobin than the three *D. pinodes* baseline isolates. In addition, 96% of North Dakota isolates were less sensitive to the fungicide than the previously established baseline (Bowness et al., 2016). No baseline is available for *A. pisi*; therefore, our comparisons were made to two isolates previously determined to be sensitive to pyraclostrobin and again, nearly all isolates collected in 2019 and 2020 displayed lower sensitivities. The *in vitro* results were well-supported by greenhouse evaluations conducted across a series of 10-fold fungicide dilutions. These findings have prompted recommendations to growers that they discontinue the use of QoI fungicides for the management of Ascochyta blight in field peas.

The first field observations in North Dakota suggesting reduction in efficacy and control for pyraclostrobin occurred in 2017; however, it is unclear when resistance first developed. Results from a previous study indicated that 6% of the 304 *D. pinodes* isolates collected in 2010 and 2011 were highly insensitive to pyraclostrobin (Bowness et al., 2016). No isolates collected in the US displayed insensitivity, including isolates collected from North Dakota in 2010 and 2011. Insensitive *D. pinodes* isolates represented populations that potentially were exposed to the class of chemistry for up to eight years in Alberta and Saskatchewan. The shift in *in vitro* sensitivity was supported by in planta studies (Bowness et al., 2016). No reduced-sensitivity or resistance to pyraclostrobin was detected among 810 A*. pisi* isolates collected from 2014 to 2016 from a survey of grower fields and seed lots in Montana (Owati et al., 2017).

The Rf for *D. pinodes* and *A. pisi* isolates evaluated in this study to pyraclostrobin ranged from 2.4 to 1,428.6, and 1.2 to 208.3, respectively. A greater than 500-fold shift in sensitivity in some *D. pinodes* isolates and 36% of isolates displaying EC_50_ values >100 µg/ml lead to the hypothesis that these isolates contain the G143A mutation (Gisi et al., 2002; Wise et al., 2009; Owati et al., 2017). A similar situation was observed with *A. pisi* isolates where 18% of isolates displayed EC_50_ values >100 µg/ml pyraclostrobin. The large observed range in Rf suggests that the F129L mutation also may be present in some isolates evaluated (Pasche et al., 2004; Pasche and Gudmestad, 2008). Significant differences in control by pyraclostrobin observed among reduced-sensitive/resistant isolates of *D. pinodes* and *A. pisi* under greenhouse conditions support the hypothesis that both the F129L and G143A mutations may be present. The presence of both mutations has been reported previously in *Pyricularia grisea* and *Pyrenophora tritici-repentis* (Kim et al., 2003; Sierotzki et al., 2007). Therefore, further work is important to determine the mechanism of reduced sensitivity/resistance in these two Ascochyta blight pathogens.

Little research has been conducted evaluating sensitivity of Ascochyta blight pathogens to prothioconazole. A prothioconazole sensitivity monitoring program was established in North Dakota due to the detection of QoI reduced sensitivity/resistance. Baseline isolates of *D. pinodes* and known sensitive isolates of *A. pisi* evaluated in the current study displayed relatively low levels of variability in response to prothioconazole, with the difference between the most- and least-sensitive isolates 3.2- and 4.1-fold, respectively. High levels of variability in prothioconazole response were reported for 29 *D. pinodes* isolates collected from the Pacific Northwest region of the US and 23 isolates collected from Alberta and Saskatchewan (Delgado et al., 2011). A bimodal pattern of sensitivity, as was observed with these baseline isolates, can result from natural variability in isolate sensitivity, or represent distinct fungicide sensitive and insensitive populations (Brent and Hollomon, 2007). However, by definition, these baseline *D. pinodes* isolates had no previous exposure to any DMI fungicide (Delgado et al., 2011). Prothioconazole sensitivity of 30 *D. pinodes* isolates collected from northeastern Montana in 2012 was similar to that observed for the most sensitive isolates of the baseline population, despite potentially being to exposed to the fungicide (Delgado et al., 2011; Lonergan et al., 2015). Sensitivity of a majority (82%) of *D. pinodes* isolates collected in North Dakota from 2017 to 2020 fell within the previously established baseline for prothioconazole (Delgado et al., 2011). However, 83% were less sensitive than the 2012 *D. pinodes* isolates from Montana which displayed a much narrower range in sensitivity (Lonergan et al., 2015). More than 90% of *D. pinodes* and *A. pisi* isolates collected from 2017 to 2020 were less sensitive to prothioconazole than the baseline/known sensitive isolates tested within our study. This suggests either the presence of a naturally occurring prothioconazole reduced-sensitive and/or resistant *D. pinodes* and *A. pisi* pathogen population in North Dakota, or more likely, that isolates may have been exposed to prothioconazole (Damicone and Smith, 2009). Previous studies reported *A. rabiei* isolates exposed to prothioconazole for as little as one growing season exhibited a significant decrease in *in vitro* sensitivity when compared to the baseline isolates (Wise et al., 2011). We are not aware of previous research studies assessing *in vitro* fungicide sensitivity of *A. pisi* to prothioconazole.

Previous studies suggest that *in vitro* sensitivity assays are useful in predicting shifts in fungicide efficacy, but it is necessary to conduct *in vivo* studies on a subset of isolates with representative *in vitro* sensitivities to confirm that *in vitro* shifts accurately correspond to reductions in disease control (Wise et al., 2009). While sensitivity of one *D. pinodes* isolate from Zhejiang Province, China aligned with reduced-sensitive isolates evaluated in the current study, *in vitro* results were not supported by *in vivo* fungicide efficacy evaluations in that study (Liu et al., 2016). In the greenhouse studies performed here, disease control provided by pyraclostrobin and prothioconazole was significantly lower in isolates of *D. pinodes* and *A. pisi* displaying reduced-sensitivity/resistance in *in vitro* assays when compared to sensitive. A 5- to 10-fold shift in *in vitro* sensitivity resulted in a significant reduction in disease control provided by both fungicides under greenhouse conditions. When pyraclostrobin was applied at the highest concentration (100 µg/ml), reductions in disease severity on plants inoculated with *D. pinodes* isolates with *in vitro* EC_50_ values of >100 µg/ml were 32 and 34%, which are commercially unacceptable levels of disease control (Wise et al., 2009). The relationship between *in vitro* and *in vivo* results is supported by the regression analyses. However, these results do not confirm a significant shift in prothioconazole sensitivity is occurring in North Dakota. To date, no loss of disease control provided by that fungicide has been reported from field pea production fields in the state.

## 5 Conclusions

Ascochyta blight of field pea can be devastating when the disease is inadequately managed. The results reported here reveal the diversity of pathogen populations of the Ascochyta blight complex of field pea present in North Dakota along with a shift in sensitivity of *D. pinodes* and *A. pisi* to pyraclostrobin and prothioconazole. Despite the presence of *A. pisi* isolates displaying similar aggressiveness to *D. pinodes* isolates, *D. pinodes* is the most abundant species in the Ascochyta blight pathogen complex of field pea in the state and is likely the most important pathogen causing Ascochyta blight. *D. pinodes* is considered high-risk pathogen for fungicide resistance development due to sexual recombination that increases variation in morphology, physiology, and aggressiveness. A large majority of *D. pinodes* and *A. pisi* isolates collected during this survey displayed some level of reduced-sensitivity or resistance to both fungicides in *in vitro* and greenhouse assays. Reductions in control of Ascochyta blight in field peas by pyraclostrobin have been reported, but no such observations have been made for prothioconazole. Therefore, growers should discontinue the use of pyraclostrobin for the management of Ascochyta blight. Results of this study provide the foundation for the development of timely, relevant and economically-viable disease management recommendations that will limit yield loss, guide future research, and potentially delay resistance development to the limited number of fungicides available to field pea producers in North Dakota.

## Data availability statement

The original contributions presented in the study are included in the article/**Supplementary Material**. Further inquiries can be directed to the corresponding author.

## Author contributions

DF: methodology, investigation, data curation, writing - review & editing. SM: writing – review & editing. MZ: statistical analyses – review & editing. ME: writing – review & editing. JP: writing – review & editing, supervision, project administration, funding procurement. All authors contributed to the article and approved the submitted version.
