# Supplementary material for: Ascochyta blight in North Dakota field pea: the pathogen complex and its fungicide sensitivity
Source: Front Plant Sci. 2023 Aug 2;14:1165269. doi: 10.3389/fpls.2023.1165269 (PMC10434212; doi:10.3389/fpls.2023.1165269)
Supplement: Supplementary file 1 [file Table_1.pdf]

Supplementary Table S1. Collection data and effective concentration at which the mycelial growth is inhibited by 50% (EC50) of *Didymella pinodes* isolates used in in vitro fungicide sensitivity assays from 2001 to 2020.

				EC_{50} (µg/ml)			
				Pyraclostrobin		Prothioconazole	
Year	Isolate	State	County	Mean	Std dev	Mean	Std dev
2001	Ap-1[a]	ND	Unknown	0.04	0.005	0.10	0.015
2001	Ap-8[a]	ND	Unknown	0.05	0.005	0.32	0.024
2001	Mp-1[a]	WA	Unknown	0.12	0.008	0.18	0.017
2017	C-1	ND	Foster	>100	0.000	6.06	0.983
2017	C-2	ND	Foster	35.71	0.082	51.17	2.827
2017	C-4	ND	Foster	>100	0.000	0.93	0.166
2017	C-5	ND	Foster	>100	0.000	6.70	0.207
2017	C-6	ND	Foster	>100	0.000	2.11	0.719
2017	L-2	ND	Cavalier	6.23	0.008	3.25	0.691
2017	L-3	ND	Cavalier	19.70	0.091	0.21	0.017
2017	2-3	ND	Cavalier	13.43	0.016	0.83	0.183
2017	3-1	ND	Cavalier	>100	0.000	0.36	0.225
2017	3-2	ND	Cavalier	19.47	0.024	11.85	0.464
2017	3-3	ND	Cavalier	>100	0.000	1.01	0.221
2018	T1R2-1	ND	Foster	23.63	0.017	20.91	1.144
2018	T1R2-6	ND	Foster	0.27	0.024	12.33	1.789
2018	T2R2-5	ND	Foster	40.51	0.075	28.15	4.757
2018	T2R2-7	ND	Foster	>100	0.000	21.61	1.640
2018	T2R3-9	ND	Foster	0.27	0.000	18.50	2.219
2018	T2R4-4	ND	Foster	49.21	8.106	40.93	2.058
2018	T2R4-8	ND	Foster	>100	0.000	2.02	0.521
2018	T2R4-11	ND	Foster	52.47	4.342	33.95	2.739
2018	T2R4-12	ND	Foster	78.42	7.991	45.73	5.804
2018	T2R5-1	ND	Foster	0.18	0.062	1.84	0.277
2018	T2R5-2	ND	Foster	0.28	0.096	5.07	0.559
2019	1-1	ND	Cavalier	>100	0.000	6.63	0.793
2019	1-3	ND	Cavalier	89.13	4.569	5.14	0.760
2019	3-1	ND	Rolette	39.20	3.138	4.15	0.585
2019	3-2	ND	Rolette	1.92	0.430	5.52	0.874
2019	3-4	ND	Rolette	64.81	7.504	3.64	1.977
2019	3-5	ND	Rolette	2.24	0.746	5.35	1.982
2019	4-1	ND	Rolette	2.21	1.231	0.15	0.016
2019	4-3	ND	Rolette	>100	0.000	3.00	0.095
2019	5-1	ND	Rolette	82.78	6.601	0.50	0.017
2019	5-2	ND	Rolette	3.62	1.216	0.10	0.018
2019	5-4	ND	Rolette	>100	0.000	0.75	0.017
2019	6-1	ND	McHenry	0.55	0.173	4.32	0.410
2019	7-1	ND	Mountrail	>100	0.000	5.55	0.250
2019	7-2	ND	Mountrail	0.97	0.242	10.05	1.665
2019	8-1	ND	Mountrail	>100	0.000	3.60	0.198

				EC$_{50}$ (µg/ml)			
				Pyraclostrobin		Prothioconazole	
Year	Isolate	State	County	Mean	Std dev	Mean	Std dev
2019	8-4	ND	Mountrail	3.14	0.332	7.20	0.713
2019	9-3	ND	Mountrail	22.74	0.163	2.15	0.088
2019	9-4	ND	Mountrail	>100	0.000	7.15	0.779
2019	9-5	ND	Mountrail	>100	0.000	5.00	0.573
2019	10-1	ND	Mountrail	1.93	0.286	6.12	0.105
2019	10-2	ND	Mountrail	77.25	2.500	0.10	0.018
2019	10-5	ND	Mountrail	65.06	16.803	0.87	0.376
2019	11-1	ND	Mountrail	83.47	4.550	1.84	0.239
2019	11-2	ND	Mountrail	>100	0.000	2.84	0.379
2019	11-3	ND	Mountrail	1.38	1.229	4.25	0.690
2019	11-4	ND	Mountrail	85.28	6.416	3.15	0.169
2019	11-5	ND	Mountrail	>100	0.000	4.45	0.234
2019	13-1	ND	McLean	>100	0.000	4.00	0.351
2019	13-3	ND	McLean	66.51	5.483	1.14	0.049
2019	13-5	ND	McLean	2.01	0.657	3.55	0.156
2019	14-1	ND	McLean	6.01	0.841	6.47	0.076
2019	14-2	ND	McLean	10.50	0.577	3.47	0.260
2019	15-1	ND	Ward	>100	0.000	5.81	1.038
2019	15-2	ND	Ward	9.47	0.511	5.00	0.392
2019	18-1	ND	Burleigh	1.74	0.774	0.20	0.029
2019	18-3	ND	Burleigh	2.24	0.824	4.00	0.626
2019	19-4	ND	Wells	69.41	1.078	1.74	0.585
2019	20-1	ND	Cass	1.26	0.303	4.89	0.232
2019	20-2	ND	Cass	5.37	0.283	3.91	0.174
2019	20-3	ND	Cass	5.60	0.121	6.30	0.176
2019	21-2	ND	Cass	1.50	0.875	6.09	0.403
2019	22-3	ND	Cass	>100	0.000	7.15	0.380
2019	24-1	ND	Burke	3.37	0.221	5.15	0.175
2019	25-1	ND	Ward	>100	0.000	6.11	0.688
2019	25-3	ND	Ward	10.70	5.723	5.03	0.387
2019	25-5	ND	Ward	>100	0.000	4.98	0.373
2019	27-1	ND	Foster	88.74	13.438	0.80	0.099
2019	27-5	ND	Foster	>100	0.000	4.13	0.448
2019	28-2	ND	Foster	1.16	0.226	2.44	0.052
2019	28-4	ND	Foster	81.87	8.924	3.44	0.305
2019	29-2	ND	Foster	2.59	0.037	8.25	0.445
2019	29-3	ND	Foster	>100	0.000	6.50	0.544
2019	29-5	ND	Foster	>100	0.000	3.99	0.556
2019	30-1	ND	Foster	5.03	3.807	1.40	0.091
2019	31-3	ND	Foster	0.17	0.015	0.34	0.014
2019	31-5	ND	Foster	>100	0.000	0.41	0.033
2019	32-1	ND	McKenzie	1.47	0.732	6.13	0.411
2019	32-5	ND	McKenzie	1.16	0.236	5.13	0.532

				EC_{50} (µg/ml)			
				Pyraclostrobin		Prothioconazole	
Year	Isolate	State	County	Mean	Std dev	Mean	Std dev
2019	33-3	ND	Walsh	0.19	0.162	10.00	1.570
2019	33-5	ND	Walsh	2.81	0.495	4.20	0.349
2019	34-1	ND	Walsh	6.78	4.601	36.05	1.193
2019	35-1	ND	Walsh	4.55	1.386	0.98	0.022
2019	36-2	ND	Cavalier	>100	0.000	2.00	0.084
2019	36-3	ND	Cavalier	87.83	9.170	2.15	0.187
2019	36-4	ND	Cavalier	1.93	0.818	0.20	0.114
2019	36-5	ND	Cavalier	1.19	1.038	0.25	0.013
2019	37-1	ND	Cass	6.03	1.043	4.15	0.177
2019	37-2	ND	Cass	>100	0.000	6.25	0.264
2019	38-1	ND	Cass	>100	0.000	5.70	0.222
2020	39-1	ND	Cavalier	>100	0.000	1.81	0.125
2020	39-2	ND	Cavalier	>100	0.000	6.11	1.107
2020	39-3	ND	Cavalier	>100	0.000	0.26	0.188
2020	39-4	ND	Cavalier	>100	0.000	60.91	9.507
2020	40-1	ND	Cavalier	1.14	0.308	23.99	3.806
2020	40-2	ND	Cavalier	>100	0.000	24.89	0.235
2020	40-4	ND	Cavalier	>100	0.000	0.19	0.058
2020	41-1	ND	Cavalier	>100	0.000	1.18	0.050
2020	41-2	ND	Cavalier	>100	0.000	1.75	0.179
2020	41-3	ND	Cavalier	>100	0.000	3.34	0.582
2020	41-4	ND	Cavalier	>100	0.000	16.97	1.289
2020	42-1	ND	Cavalier	75.13	0.000	5.78	0.626
2020	45-1	ND	Ward	>100	0.000	2.17	0.557
2020	45-2	ND	Ward	0.34	0.078	0.47	0.078
2020	46-2	ND	Ward	>100	0.000	1.39	0.276
2020	47-2	ND	Burke	6.34	9.721	3.64	0.448
2020	48-1	ND	Burke	>100	0.000	1.83	0.205
2020	48-2	ND	Burke	>100	0.000	4.22	0.256
2020	48-3	ND	Burke	>100	0.000	0.67	0.359
2020	48-4	ND	Burke	>100	0.000	1.76	0.201
2020	49-1	ND	Williams	>100	0.000	2.61	0.437
2020	49-2	ND	Williams	>100	0.000	4.02	0.269
2020	51-1	ND	Mountrail	>100	0.000	1.16	0.349
2020	51-2	ND	Mountrail	4.46	0.184	2.24	0.696
2020	51-3	ND	Mountrail	>100	0.000	1.05	0.312
2020	51-4	ND	Mountrail	6.94	1.929	34.88	5.032
2020	52-1	ND	Foster	3.24	0.607	0.16	0.013
2020	52-3	ND	Foster	3.65	0.188	67.58	6.063
2020	53-1	ND	Foster	3.95	0.583	0.64	0.412
2020	53-2	ND	Foster	3.25	1.422	0.78	0.213
2020	53-3	ND	Foster	3.85	0.409	0.14	0.045
2020	53-4	ND	Foster	0.44	0.064	8.10	0.626

				EC$_{50}$ (µg/ml)			
				Pyraclostrobin		Prothioconazole	
Year	Isolate	State	County	Mean	Std dev	Mean	Std dev
2020	54-1	ND	Cavalier	4.41	0.480	7.24	0.597
2020	54-2	ND	Cavalier	2.21	1.360	5.96	0.624
2020	54-3	ND	Cavalier	4.04	0.443	21.37	0.236
2020	54-4	ND	Cavalier	3.87	0.262	0.17	0.013
2020	55-1	ND	Cavalier	3.77	0.750	13.89	1.558
2020	55-2	ND	Cavalier	3.69	0.454	13.89	3.178
2020	55-3	ND	Cavalier	3.57	0.225	21.83	2.793
2020	56-1	ND	Cavalier	3.89	0.773	23.57	2.398
2020	56-2	ND	Cavalier	4.14	0.389	26.81	0.604
2020	56-3	ND	Cavalier	4.24	0.189	2.56	0.196
2020	56-4	ND	Cavalier	4.69	0.189	11.42	2.013
2020	56-5	ND	Cavalier	4.27	0.375	4.01	0.226

[a]Baseline isolates, collected prior to fungicide registration (Delgado et al., 2011).
