# Supplementary material for: Ascochyta blight in North Dakota field pea: the pathogen complex and its fungicide sensitivity
Source: Front Plant Sci. 2023 Aug 2;14:1165269. doi: 10.3389/fpls.2023.1165269 (PMC10434212; doi:10.3389/fpls.2023.1165269)
Supplement: Supplementary file 2 [file Table_2.pdf]

Supplementary Table S2. Collection data and effective concentration at which the mycelial growth is inhibited by 50% (EC50) of *Ascochyta pisi* isolates used in in vitro fungicide sensitivity assays from 2015 to 2020.

				EC_{50} (µg/ml)			
				Pyraclostrobin		Prothioconazole	
Year	Isolate	State	County	Mean	Std dev	Mean	Std dev
2015	D2[a]	MT	Unknown	0.61	0.037	0.61	0.017
2016	Dp-3[a]	MT	Unknown	0.35	0.024	0.15	0.022
2019	1-5	ND	Cavalier	0.90	0.106	7.63	1.063
2019	2-2	ND	Cavalier	0.94	0.085	6.93	0.917
2019	2-3	ND	Cavalier	1.49	0.185	7.74	0.841
2019	3-3	ND	Rolette	1.08	0.142	5.15	0.668
2019	4-2	ND	Rolette	>100	0.000	4.13	0.083
2019	4-4	ND	Rolette	2.24	0.024	2.13	0.070
2019	6-2	ND	McHenry	0.94	0.054	7.66	1.049
2019	6-3	ND	McHenry	18.98	0.260	4.63	0.365
2019	7-3	ND	Mountrail	0.95	0.019	5.02	0.583
2019	8-3	ND	Mountrail	54.15	2.452	4.30	0.159
2019	9-2	ND	Mountrail	>100	0.000	5.00	0.408
2019	10-3	ND	Mountrail	3.39	0.088	2.53	1.150
2019	10-4	ND	Mountrail	4.15	0.806	0.97	0.039
2019	12-3	ND	Mountrail	1.38	0.580	5.48	1.554
2019	12-5	ND	Mountrail	0.95	0.172	7.09	0.867
2019	13-2	ND	McLean	>100	0.000	2.14	0.098
2019	13-4	ND	McLean	1.65	0.244	3.91	0.720
2019	14-3	ND	McLean	1.25	0.096	7.47	0.487
2019	15-3	ND	Ward	2.84	0.093	5.99	0.456
2019	15-4	ND	Ward	1.51	0.317	6.81	1.444
2019	17-1	ND	McLean	1.44	0.132	5.06	1.901
2019	17-2	ND	McLean	28.82	0.166	4.06	0.079
2019	17-3	ND	McLean	>100	0.000	5.56	0.327
2019	17-4	ND	McLean	>100	0.000	5.75	0.102
2019	18-2	ND	Burleigh	1.90	0.044	6.58	0.347
2019	19-2	ND	Wells	2.00	0.236	5.05	0.684
2019	19-5	ND	Wells	1.97	0.300	5.60	0.983
2019	20-4	ND	Cass	0.56	0.033	3.15	0.095
2019	21-4	ND	Cass	>100	0.000	2.95	0.048
2019	22-1	ND	Cass	39.52	0.818	3.00	0.091
2019	23-1	ND	Cass	2.02	0.693	4.89	0.956
2019	24-2	ND	Burke	1.93	0.277	5.72	0.465
2019	24-3	ND	Burke	2.21	0.430	5.02	1.396
2019	25-2	ND	Ward	3.45	1.224	3.31	0.033
2019	25-4	ND	Ward	74.69	2.332	5.31	0.077
2019	26-1	ND	Foster	1.39	0.411	5.31	0.362
2019	26-3	ND	Foster	69.49	5.161	1.18	0.062
2019	27-2	ND	Foster	78.99	8.052	0.80	0.268

Year	Isolate	State	County	EC$_{50}$ (µg/ml)			
				Pyraclostrobin		Prothioconazole	
				Mean	Std dev	Mean	Std dev
2019	27-3	ND	Foster	2.16	0.289	4.68	0.762
2019	30-2	ND	Foster	1.43	1.123	2.40	0.185
2019	30-3	ND	Foster	>100	0.000	0.34	0.085
2019	30-5	ND	Foster	>100	0.000	3.02	0.694
2019	31-2	ND	Foster	1.25	0.468	10.00	0.147
2019	35-2	ND	Walsh	1.84	0.413	0.76	0.108
2020	40-3	ND	Cavalier	4.40	0.547	4.36	0.161
2020	46-1	ND	Ward	1.96	0.803	43.23	3.002
2020	47-1	ND	Burke	>100	0.000	4.37	0.627
2020	52-2	ND	Foster	4.09	1.227	7.08	0.104
2020	57-1	ND	Cavalier	2.36	1.751	24.54	2.117
2020	57-2	ND	Cavalier	4.62	0.959	48.26	1.486

[a]Determined to be sensitive to pyraclostrobin in previous studies (Owati et al., 2017).
